# Utilization of deep learning to quantify fluid volume of neovascular age-related macular degeneration patients based on swept-source OCT imaging: The ONTARIO study

**DOI:** 10.1371/journal.pone.0262111

**Published:** 2022-02-14

**Authors:** Simrat K. Sodhi, Austin Pereira, Jonathan D. Oakley, John Golding, Carmelina Trimboli, Daniel B. Russakoff, Netan Choudhry

**Affiliations:** 1 School of Clinical Medicine, University of Cambridge, Cambridge, United Kingdom; 2 Department of Ophthalmology & Visual Sciences, University of Toronto, Toronto, ON Canada; 3 Voxeleron LLC, San Francisco, United States of America; 4 Vitreous Retina Macula Specialists of Toronto, Etobicoke, ON, Canada; 5 Cleveland Clinic Canada, Toronto, ON, Canada; Massachusetts Eye & Ear Infirmary, Harvard Medical School, UNITED STATES

## Abstract

**Purpose:**

To evaluate the predictive ability of a deep learning-based algorithm to determine long-term best-corrected distance visual acuity (BCVA) outcomes in neovascular age-related macular degeneration (nARMD) patients using baseline swept-source optical coherence tomography (SS-OCT) and OCT-angiography (OCT-A) data.

**Methods:**

In this phase IV, retrospective, proof of concept, single center study, SS-OCT data from 17 previously treated nARMD eyes was used to assess retinal layer thicknesses, as well as quantify intraretinal fluid (IRF), subretinal fluid (SRF), and serous pigment epithelium detachments (PEDs) using a novel deep learning-based, macular fluid segmentation algorithm. Baseline OCT and OCT-A morphological features and fluid measurements were correlated using the Pearson correlation coefficient (PCC) to changes in BCVA from baseline to week 52.

**Results:**

Total retinal fluid (IRF, SRF and PED) volume at baseline had the strongest correlation to improvement in BCVA at month 12 (PCC = 0.652, p = 0.005). Fluid was subsequently sub-categorized into IRF, SRF and PED, with PED volume having the next highest correlation (PCC = 0.648, p = 0.005) to BCVA improvement. Average total retinal thickness in isolation demonstrated poor correlation (PCC = 0.334, p = 0.189). When two features, mean choroidal neovascular membranes (CNVM) size and total fluid volume, were combined and correlated with visual outcomes, the highest correlation increased to PCC = 0.695 (p = 0.002).

**Conclusions:**

In isolation, total fluid volume most closely correlates with change in BCVA values between baseline and week 52. In combination with complimentary information from OCT-A, an improvement in the linear correlation score was observed. Average total retinal thickness provided a lower correlation, and thus provides a lower predictive outcome than alternative metrics assessed. Clinically, a machine-learning approach to analyzing fluid metrics in combination with lesion size may provide an advantage in personalizing therapy and predicting BCVA outcomes at week 52.

## Introduction

Age-related macular degeneration (ARMD) remains the leading cause of blindness worldwide, with the prevalence expected to rise to 288 million by 2040 [1]. The mainstay of treatment for neovascular ARMD (nARMD) remains anti-vascular endothelial growth factor (VEGF) intravitreal injections due to the high efficacy and tolerable safety profile demonstrated in clinical trials [2, 3]. A recent meta-analysis found an overall increase of 7.37 letters in best corrected visual acuity (BCVA) at 1-year following intravitreal aflibercept treatment [4]. BCVA remains the most robust prognostic consideration in nARMD treatment trials [5]. Optical coherence tomography (OCT) imaging has allowed for anatomical assessment and correlation to treatment progression [6]. However, measurements used in initial pivotal clinical trials, such as central subfield thickness (CST), have recently demonstrated poor correlation to BCVA outcomes [7]. Fluid analysis of intraretinal cystoid fluid (IRF), subretinal fluid (SRF) and pigment-epithelial detachments (PED) have demonstrated promise in predicting functional deficits in nARMD, but currently lack consistent correlative results [8]. With patient demand increasing for accurate predictions in treatment outcomes, more diverse imaging biomarkers are crucial for providing accurate prognostic information.

Optical coherence tomography angiography (OCT-A) has revealed further retinal anatomical and vascular structures to track nARMD progression [9]. Our 2021 proof of concept CANADA trial demonstrated significantly improved choroidal neovascularization membrane (CNVM) size identification for nARMD treatment monitoring through OCT-A [10]. However, fluid volume metrics and delineation of unique fluid characterization required extensive manual segmentation and demonstrated poor acquisition of real-time values [10]. It was hypothesized that artificial intelligence (AI) would improve image segmentation, ultimately providing better personalized therapy.

Machine learning has bridged the gap in OCT imaging interpretation of biomarkers and nARMD disease monitoring. Rohm et al. in 2018 found that deep learning networks showed good visual predictions at 3-months following anti-VEGF treatment for nARMD [11]. Schlegl et al. in 2018 found deep learning networks in OCT imaging analysis led to excellent accuracy in retinal fluid type detection and segmentation for a variety of exudative disease processes [12]. Schmidt-Erfurth et al. in 2018 concluded that machine learning analysis of OCT images correlated with horizontal extension of IRF to final BCVA [13]. The Protocol T team also demonstrated automated segmentation of fluid on OCT imaging in diabetic macular edema patients through deep learning algorithms and concluded that SRF was associated with poor pre-treatment vision and positive anti-VEGF response [14].

While AI models have postulated promise in OCT fluid segmentation and have demonstrated predictive value in anti-VEGF treatment outcomes, there are currently no trials demonstrating machine learning with swept-source OCT images in concordance with OCT-A analysis for nARMD patients. Therefore, our study was conducted as a follow-up, phase IV, proof-of-concept trial to evaluate a novel deep learning algorithm to determine BCVA outcomes in nARMD patients using both OCT and OCT-A imaging.

## Methods

### Study design

Patients who previously completed the CANADA study were enrolled in this phase IV, retrospective, open-label proof-of-concept study. Analysis, as part of this study, began in July 2020 and ended in December 2020. The selection of participants for the CANADA study has been previously reported [10]. Twenty-three patients (25 eyes) completed the CANADA study and were enrolled into this study. No additional recruitment was performed as part of this study. This study was approved by the Institutional Review Board of Advarra and followed the tenets of the Declaration of Helsinki. Written informed consent was obtained from all participants and was in accordance with current ICH/GCP guidelines, section 4.8.10.

### Inclusion and exclusion criteria

The key inclusion criteria were: (1) ≥ 25 years of age; (2) diagnosis of nARMD; (3) treatment naïve and (4) previously enrolled in the CANADA study. Exclusion criteria included patients with uncontrolled systemic hypertension or thromboembolic events (stroke, transient ischemic attack, myocardial infarction) within 6 months from baseline, ocular conditions affecting visual acuity besides nARMD (i.e. amblyopia, ischemic optic neuropathy, clinical significant diabetic macular edema, severe non-proliferative diabetic retinopathy, glaucoma, retinal detachment, retinal dystrophies, other retinal degenerations), ocular or periocular infection or active intraocular inflammation, hypersensitivity to aflibercept or any ingredient in the formulation, previous ocular surgery (including cataract extraction or YAG capsulotomy) within 3 months from baseline, planned ocular surgery throughout the study or previous treatments of laser photocoagulation or intravitreal anti-VEGF or steroid treatments. Pregnant women, nursing women, or patients unwilling to provide informed consent were also excluded.

### Visits and treatment

Participants were not treated as part of the ONTARIO study; however, participants were enrolled from the CANADA study where they were treated with initial 3 monthly injections (loading phase) of intravitreal aflibercept 2 mg at baseline, month 1 and month 2, then every other month for a total of 12 months (52 weeks).

### Imaging

As part of the CANADA study, all patients underwent SS-OCT-A testing using the Topcon Triton Swept Source OCT (Tokyo, Japan) to identify CST and lesion size at baseline [10]. The SS-OCT-A system has a scanning speed of 100,000 A-scans per second and utilizes a wavelength-sweeping laser, with a central wavelength of 1,050 nm wavelength and a sweeping range of approximately 100 nm [15]. The OCT-A B-scans were manually segmented into 6 slabs: vitreous, superficial, outer retina (OR), full macula, deep and choriocapillaris (CC). CNVM lesion size measurements were manually performed (SS and AP) and utilized the OR and CC slabs of 6 mm x 6mm OCT-A scans. OCT-A scans with a signal to noise ratio of <7 were excluded from the study.

Baseline 7 mm x 7 mm 3D macula volume scans containing 512 A-scans and 256 B-scans were segmented using a prototype version of the Orion software (Voxeleron LLC, San Francisco, USA). This performs layer segmentation as a pre-processing step to fluid segmentation that uses a deep learning-based algorithm. Performance of the fluid segmentation has previously been validated relative to two expert graders (SS and JO) [10].

### Deep learning algorithm

**Fig 1:**
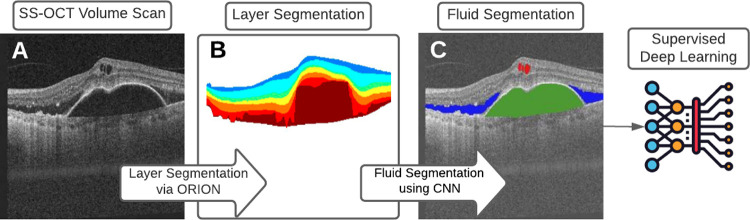
Each (A) SS-OCT volume scan is first automatically segmented using Orion into 8 retinal interfaces. This results in (B) 7 layers that can be encoded into image form and used as an additional channel in model creation, thus encoding spatial information regarding the location of (C) fluid within the retina.

A deep learning-based algorithm was implemented to segment fluid regions within each OCT-volume. As reported previously, the method takes each OCT B-scan as input, and a segmentation mask is generated automatically using Orion software (San Francisco, CA, USA) [16, 17]. The deep learning architecture used for first the training, and subsequently the testing, was that of U-Net, which is a version of the autoencoder that uses skip connections to better maintain detail across different scales [18, 19]. U-Net uses three encoding/decoding levels, and learning was based on minimizing the model’s loss, where the loss function combines categorical cross entropy (CCE) and a weighted Dice similarity coefficient (DSC) (Fig 1).

### Statistical analysis

All data are presented as either mean or mean ± SD. The BCVA was converted to the logarithm of the minimum angle of resolution (logMAR) for statistical purposes. Counting fingers vision was given a value of 1.85, and hand motions vision was given a value of 2.30 [20]. Statistical analysis was performed within Matlab (Natwick, MA, USA) and Excel (Microsoft, Redmond, WA, USA). Pearson’s Correlation Coefficient (PCC) was used to quantify the correlation between (i) BCVA and fluid volume and (ii) BCVA and CNVM lesion size. A P-value of <0.05 was considered statistically significant. Pre-specified statistical analysis included: (1) correlation between BCVA and fluid volume at baseline and (2) correlation between BCVA and mean CNVM lesion size (mm^2^) at baseline.

## Results

### Patient characteristics

#### Fluid segmentation training

Twenty-three patients (25 eyes) completed the CANADA study and were enrolled in this subsequent study. One patient was excluded based on image quality and subsequent layer segmentation issues. Therefore, the final cohort consisted of 22 patients (24 eyes). The average age of the patients was 76 years (range 48–89 years). Fifteen of the 22 treated patients were female and 7 of the 22 treated patients were male. Fifteen of the 24 eyes were pseudophakic and 2 of 24 eyes were phakic at baseline and were pseudophakic at week 52. For the entire cohort, the mean BCVA at baseline was 20/125 and improved to 20/80 by week 52 (p<0.001).

Twenty-two SS-OCT volumes of the macula, comprising 5,632 images from the 22 nARMD subjects in the final cohort, were used to quantify IRF, SRF and fluid in serous PEDs. Assessment used 10-fold cross-validation, where each fold ensured no subject eye data was in both the training and testing data. In each fold, the training set used data augmentation such that one volume, comprising 256 B-scans, was replicated six times using random scaling, rotations and shifting. Given these were large input volumes and a heterogeneous input data set, the reported validation compared excellently to manual segmentations. Results showed strong correlation in all fluid volumes between the algorithm and the manually labeled data [17].

#### Predictive analysis subgroup

OCT-A analysis was performed as part of the CANADA study to determine the mean CNVM lesion size (μm^2^). Of the final cohort of 22 patients (24 eyes) used in the fluid segmentation, 17 patients (18 eyes) had OCT-A data due to data or segmentation issues in 5 patients [10]. There were no serious adverse events throughout the course of the study. The mean age in this subgroup was consistent with the full cohort at 79 years (± 7.02). The mean BCVA at baseline was 20/125 and increased to 20/80 by week 52 (p = 0.0141).

### Fluid analysis

Total fluid volume at baseline and change in logMAR at month 12 relative to baseline had the closest correlation (PCC = 0.652, p = 0.005) (Table 1). Fluid was subsequently sub-categorized into IRF, SRF and PED, with PED volume having the next highest correlation (PCC = 0.648, p = 0.005). Average total retinal thickness in isolation gave a lower correlation (PCC = 0.334, p = 0.189), and mean CNVM size (um^2^) from 3 mm OCT-A scans was very low (PCC = 0.072, p = 0.784). When two features were combined and correlated with visual outcomes, the best correlation increased to PCC = 0.695 (p = 0.002) using mean CNVM size and total fluid volume (Table 2).

**Table 1:** The top 10 correlating features to logMar change when ranked based on PCC.

Feature 1	Pearson’s Correlation Coefficient	p-value
Total Fluid	0.6521	0.0046
PED	0.6481	0.0049
SRF	0.4824	0.0499
6 mm average CST	0.3344	0.1895
Density map–central–% 6 mm	0.3241	0.2045
6 mm inferior CST	0.3174	0.2145
6 mm nasal	0.3122	0.2225
6 mm temporal	0.2820	0.2728
Density map–superior–% 6 mm	0.2522	0.3288
6 mm superior	0.2108	0.4168

**Table 2:** The top 10 pairwise correlating features to logMar change when ranked based on PCC.

Feature 1	Feature 2	Pearson’s Correlation Coefficient	p-value
CNVM Mean size (μm^2^)– 3 mm OCTA	Total Fluid	0.6951	0.0099
PED	IRF	0.6752	0.0141
CNVM Mean size (μm^2^)– 6 mm OCTA	Total Fluid	0.6751	0.0141
CNVM Mean size (μm^2^)– 3 mm OCTA	PED	0.6721	0.0149
Total Fluid	SRF	0.6690	0.0157
Density map–inferior–% 6 mm	PED	0.6669	0.0163
Density map–superior–% 6 mm	Total Fluid	0.6659	0.0165
Total Fluid	IRF	0.6634	0.0172
Density map–inferior–% 6 mm	Total Fluid	0.6634	0.0172
Density map–central–% 6 mm	PED	0.6606	0.0181

**Fig 2:**
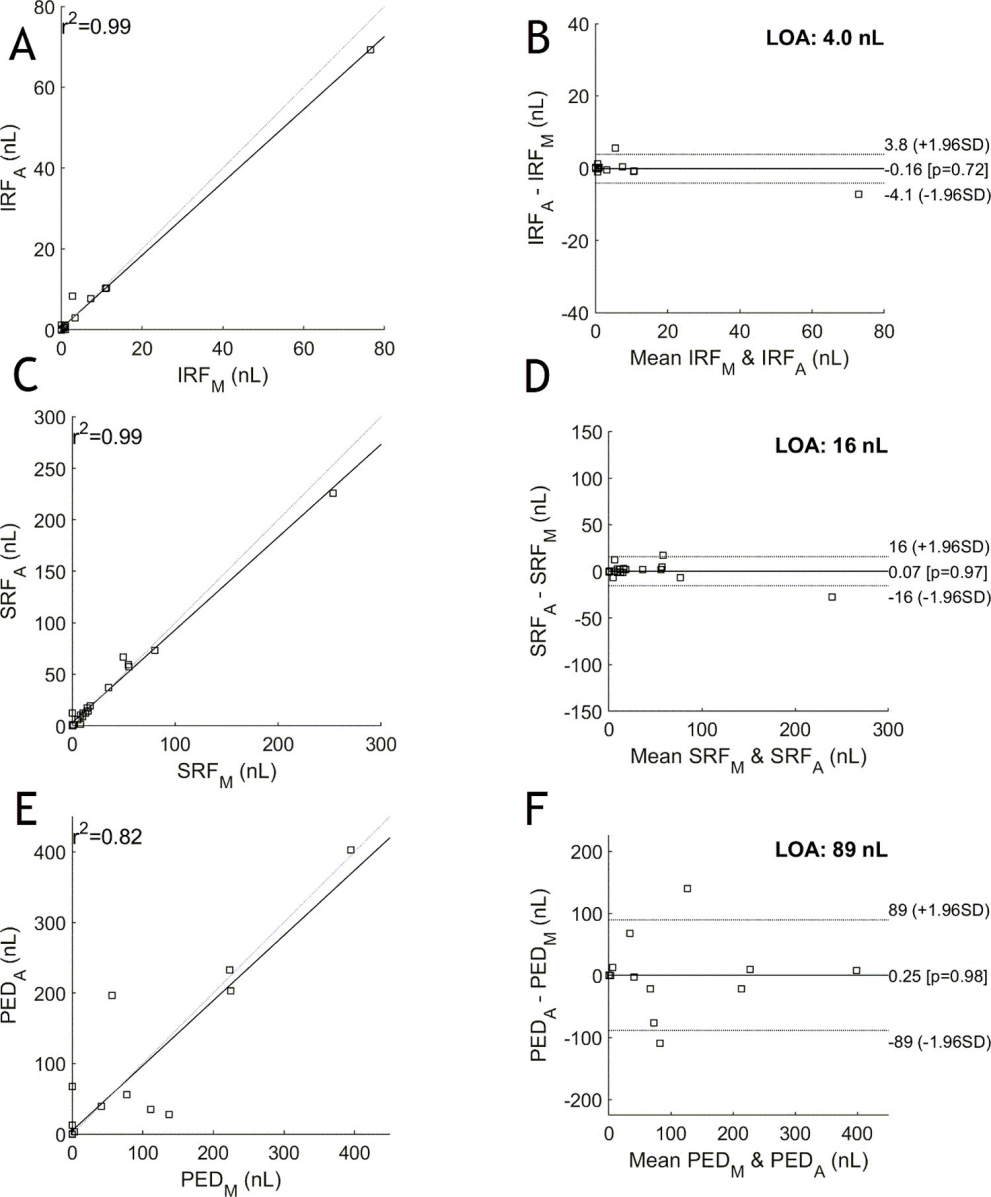
The manual versus automated reported total areas for each fluid type across all volumes on the left for IRF (A), SRF (C) and PED (E); and their corresponding Bland-Altman plots on the right for IRF (B), SRF (D) and PED (F). The correlation scores are 0.992, 0.986 and 0.820 for IRF, SRF and PED, respectively. For the Bland-Altman plots, the manual values are denoted with subscript ‘M’ and the automated values with ‘A’. Narrow limits of agreement are shown for IRF and SRF, but are larger for PED, as is addressed in the discussion.

Bland-Altman plots were used to assess the differences in agreement. In looking at the limits of agreement (LOA) in the Bland-Altman plots, we can evaluate the bias between the mean differences. Fig 2 indicates narrow limits of agreement for both IRF and SRF. Fluid due to PED, however, shows wide LOA.

## Discussion

The use of AI to automate the analysis of ocular images and allow quantification of retinal biomarkers has increased in popularity globally. Previous studies in nARMD have used spectral domain OCT (SD-OCT) to quantify fluid and used this data to predict BCVA outcomes [13]. Our study is the first to use an automated approach to segment and quantify retinal fluid in SS-OCT scans, using a novel deep learning algorithm, and combine these findings with manual OCT-A segmentation to predict BCVA outcomes at month 12. This approach showed a close correlation between total fluid volume and change in BCVA values between baseline and week 52, which was strengthened when combined with complimentary information from OCT-A.

Associating structural parameters derived from OCT data to functional outcomes has been a mainstay of clinical OCT since the ground-breaking PrONTO study [21]. Developments first in image processing techniques, and now based on deep learning, have advanced structural metrics far beyond overall retinal thickness as used in the PrONTO study. In taking advantage of such methods, we see different structure-function relationships that may be more relevant to disease type and treatment plans. In a clinical setting, it is not feasible to manually define such metrics, hence the relevance of automatic processing using AI-based methods, which allows precise outcomes without the need for manual, laborious delineation (Fig 1).

Correlation in isolation provides no information about the differences in agreement and thus additional plots need to be employed [22]. In this study, we used Bland-Altman plots to assess the limits of agreement (LOA). A wide LOA, as seen with fluid due to PED, is interpreted to be related to the challenge in delineating PEDs containing mostly fluid and those containing tissue. It is challenging for the grader to be consistent in their labelling, which results in larger discrepancies in the data, as evidenced in this plot.

The use of swept-source scans has the potential to increase the precision and recall of our automated algorithm as swept-source devices use a narrower wavelength of light and captures 100,000 A-scans per second, rather than ~68,000 A-scans that are captured by SD-OCT devices [23]. The increase in scans frequency allows for quicker image acquisition and denser scan patterns at wider fields of view than standard SD-OCT [24, 25]. This is clinically significant as it can reduce motion in the data as well as patient imaging time, improving both the quality of the data as well as the patient experience. SS-OCT and SS-OCT-A data provides extensive volumetric information and the use of AI-coupled SS-OCT-A, has the potential to become the first-line diagnostic tool in nARMD [26]. A further advantage of the SS-OCT devices is that the longer wavelength of the light source allows for deeper penetration of the choroid, resolving choroidal structures relevant to several sight-threatening diseases. Deep learning algorithms, that harness SS-OCT’s ability to penetrate the choroid, have already been developed and can segment the choroidal-scleral boundary to quantify choroidal volume [27, 28].

Due to its historical use in clinical trials, CST is currently used extensively for assessing treatment response and determining the next treatment date [2, 29]. The discrepancy between functional outcomes, measured using BCVA, and anatomical outcomes, measured using CST, is well known in the field [7]. Table 1 demonstrates a similar trend as the average total retinal thickness in isolation gave a lower correlation. Fluid volume is a more robust marker, as was shown in previous reports, as well as in our analysis [8]. When the fluid was sub-categorized into IRF, SRF and PED, the correlation was highest for PED, followed by SRF and IRF. When two parameters were combined, total fluid and CNVM mean lesion size provided the strongest correlation (PCC = 0.695, p = 0.002) to BCVA at month 12 (Table 2).

**Fig 3:**
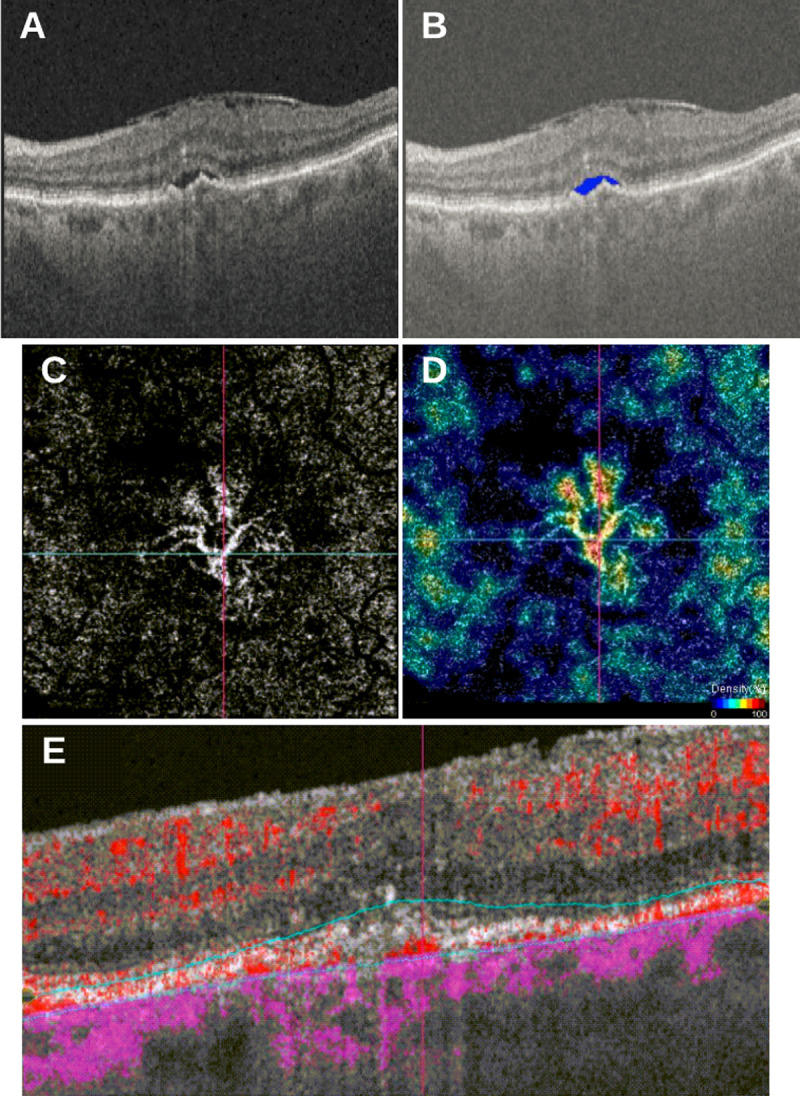
(A) SS-OCT volume scan of 87-year-old female patient with type I CNV, SRF and epiretinal membrane (ERM); (B) fluid segmentation using a convolutional neural network (CNN) highlights SRF in blue; (C) corresponding OCT-A scan depicting segmented CNVM from outer retina (OR) slab; (D) density flow highlighting areas of increased flow; (E) corresponding flow B-scan from single horizontal B-scan through center of CNVM.

Qualitative features of OCT-A scans, including medusa form, sea-fan form, pruned vascular tree pattern, tangled network pattern and vascular loop, can be assessed without the need for adjuvant software [30, 31]. A larger sample size would assist in identifying the association of various CNVM patterns on OCT-A and their prognostic value using an AI-based approach. Quantitative measures, including total vascular area (TVA), the total area (TA) and the vascular density (VD), require add-on algorithms or time-consuming manual measurements [32, 33]. Manual measurements, such as those employed by Jia et al., involved quantification of blood flow within a CNV by multiplying the number of pixels and the pixel size after using the split-spectrum amplitude-decorrelation angiography (SSADA) algorithm to improve the signal-to-noise ratio [33]. Whereas Taibouni et al developed an automated segmentation algorithm that reduced noise and enhanced vessels by Frangi filtering [32]. Currently, an embedded, quantitative algorithm does not exist in OCT-A devices, which makes it difficult to perform these measurements in a routine clinical practice. However, based on the results of this study, it is imperative to perform these measurements as they increase the predictive outcome when correlated with OCT features (Fig 3). The addition of mean lesion size, using OCT-A, may have increased the correlation with BCVA at month 12 because OCT-A provides information on the activation of the lesion [34]. In future studies, a larger number of OCT-A parameters should be analyzed to provide a wholistic ranking of features that increase predictability of long-term visual outcomes.

Several OCT and OCT-A biomarkers have been linked to visual outcomes in nARMD, including, but not limited to, branching patterns, total number of branch points, junction density, hyperreflective foci [35–38]. In this study, we have analyzed a sub-set of these biomarkers, but others should be studied to expand the list of features that are predictive of long-term visual outcomes. In our study, PED volume and IRF were predictive of visual function, however the presence or persistence of a PED may still allow a patient to achieve a relatively fair visual acuity [39]. The predictive outcomes of specific fluid types (i.e. IRF, SRF or PED) may also vary depending on the patients included in the study. A limitation of this study was the number of patients included. To have a more robust fluid analysis, not only does the total number of patients need to be higher, but each of the fluid subtypes being evaluated needs to have a similar number of scans analyzed. Increasing the number of timepoints can also provide further information as to which biomarkers are predictive early or later in treatment. Eventually a potential “retinal calculator” could be created that allows multiple parameters can be considered at one time.

This study demonstrates the clinical implementation of a novel, deep learning-based algorithm and the importance of including OCT-A quantitative parameters to existing fluid analysis algorithms to increase the predictive power. Delineating lesion size on OCT-A scans either requires time-intensive manual segmentation or additional automated software, however the inclusion of OCT-A-related parameters may be key to accurately predicting long-term visual outcomes.
